# Breastfeeding rates are high in a prenatal community support program targeting vulnerable women and offering enhanced postnatal lactation support: a prospective cohort study

**DOI:** 10.1186/s12939-021-01386-6

**Published:** 2021-03-03

**Authors:** Jane Francis, Alison Mildon, Stacia Stewart, Bronwyn Underhill, Samantha Ismail, Erica Di Ruggiero, Valerie Tarasuk, Daniel W. Sellen, Deborah L. O’Connor

**Affiliations:** 1 Nutritional Sciences, University of Toronto, Toronto, ON Canada; 2 Translational Medicine Program, The Hospital for Sick Children, Toronto, ON Canada; 3 Health Promotion & Community Engagement, Parkdale Queen West Community Health Centre, Toronto, ON Canada; 4 Dalla Lana School of Public Health, University of Toronto, Toronto, ON Canada; 5 Joannah and Brian Lawson Centre for Child Nutrition, University of Toronto, Toronto, ON Canada; 6 Anthropology, University of Toronto, Toronto, ON Canada; 7 Pediatrics, Sinai Health, Toronto, ON Canada

**Keywords:** Canada Prenatal Nutrition Program, Breastfeeding, Lactation support, Vulnerable mothers, Infant and child nutrition, Infant feeding

## Abstract

**Background:**

In Canada, 91% of all mothers initiate breastfeeding, but 40–50% stop by 6 months and only 34% breastfeed exclusively for 6 months, with lower rates among socially and/or economically vulnerable women. The Canada Prenatal Nutrition Program (CPNP) aims to support breastfeeding among vulnerable women, but there is no formal framework or funding for sites to integrate proactive postnatal breastfeeding support. This research aimed to i) describe infant feeding practices among clients of one Toronto CPNP site using charitable funds to offer a lactation support program (in-home lactation consultant visits, breast pumps); ii) determine whether breastfeeding outcomes at 6 months differ based on maternal sociodemographics and food insecurity; and iii) assess utilization of the lactation support program.

**Methods:**

Infant feeding practices were collected prospectively at 2 weeks, 2, 4 and 6 months postpartum via telephone questionnaires (*n* = 199). Maternal sociodemographics were collected at 2 weeks and food insecurity data at 6 months postpartum. Program monitoring records were used to determine utilization of the lactation support program.

**Results:**

Ninety-one percent of participants were born outside of Canada; 55% had incomes below the Low-Income Cut-Off; and 55% reported food insecurity. All participants initiated breastfeeding, 84% continued for 6 months and 16% exclusively breastfed for 6 months. Among breastfed infants, ≥76% received vitamin D supplementation. Approximately 50% of infants were introduced to solids before 6 months. Only high school education or less and food insecurity were associated with lower breastfeeding rates. Overall, 75% of participants received at least one visit with a lactation consultant and 95% of these received a breast pump.

**Conclusions:**

This study provides initial evidence that postnatal lactation support can be delivered within a CPNP site, with high uptake by clients. While all participants initiated breastfeeding and 84% continued for 6 months, adherence to the recommended 6 months of exclusive breastfeeding was low. Further research is needed to better understand the barriers to exclusive breastfeeding and how to support this practice among vulnerable women.

Study registered at clinicaltrials.gov as NCT03400605.

**Supplementary Information:**

The online version contains supplementary material available at 10.1186/s12939-021-01386-6.

**Table:** 

This article is a part of the Interventions and policy approaches to promote equity in breastfeeding collection, guest-edited by Rafael Pérez-Escamilla, PhD and Mireya Vilar-Compte, PhD	

## Background

Breastfeeding is the normal and unequalled way to feed infants, supporting optimal growth, development and health outcomes [1]. Meta-analyses have highlighted the positive associations between breastfeeding and health outcomes for both infants and mothers [2–7]. From a public health standpoint, there is a strong rationale to invest in effective breastfeeding support interventions [8].

In accordance with World Health Organization (WHO) guidance, Health Canada recommends that infants receive exclusive breastfeeding (EBF) for their first six months of life, are introduced to iron-rich complementary foods (e.g. solids) around this time and continue to be breastfed for up to two years and beyond [9, 10]. Vitamin D supplementation is recommended for all breastfed infants [9]. However, Canadian infant feeding practices do not fully align with these recommendations. For example, national data from the 2017–2018 Canadian Community Health Survey (CCHS) indicate that 91% of mothers initiate breastfeeding, but only 34% EBF for six months [11]. The social determinants of health negatively impact breastfeeding practices wherein vulnerable women (e.g. socially and/or economically disadvantaged), including those with low income and education, single parents and adolescents, have lower breastfeeding rates [12–14]. Additionally, there is evidence from CCHS data that the duration of exclusive breastfeeding is lower among those living in food insecure households and qualitative studies also suggest that food insecurity compromises breastfeeding practices in Canada [15, 16].

The national Canada Prenatal Nutrition Program (CPNP) aims to improve birth outcomes and promote and support breastfeeding among vulnerable women [17, 18]. Established in 1994, the CPNP provides federal funding for community agencies to implement maternal-infant health interventions for vulnerable pregnant women, including, for example, those with lower income/education and/or who are substance users, newcomers to Canada (immigrants or refugees), lone-parents and adolescents [17]. There are approximately 240 CPNP sites across Canada, serving over 45,000 women each year [17]. All sites are unique as they identify their own support priorities based on the communities they serve, but core services include: i) group education on nutrition and health; ii) provision of food and/or grocery gift cards; and iii) individual support and community referrals to address a variety of health and social needs.

There is limited evaluation of the CPNP at the national level and within individual sites. There are also limited data available on infant feeding practices, particularly breastfeeding duration and exclusivity, among CPNP participants. It is encouraging that breastfeeding initiation among CPNP participants was shown in the only national impact evaluation (2002–2006) to be comparable to national rates and that women with high CPNP exposure were more likely to breastfeed to 6 weeks than those with low exposure [19, 20]. However, 60% of participants discontinued breastfeeding by six weeks postpartum [19]. Prenatal breastfeeding promotion and education are key elements of the CPNP, but there is currently no formally established framework or funding for sites to integrate proactive postnatal breastfeeding support into their programs, despite the CPNP’s aim to support breastfeeding among vulnerable women.

Access to skilled lactation support commencing shortly after birth is a key intervention for helping new mothers establish breastfeeding, build skills, resolve challenges and increase breastfeeding self-efficacy [8, 21–24]. Recent systematic reviews conclude that skilled lactation support is effective at improving breastfeeding duration and exclusivity, albeit the interventions evaluated vary widely and their effectiveness among vulnerable women has not been systematically investigated [23, 25]. WHO best practice guidelines, based on evidence from systematic reviews, recommend proactive delivery of skilled breastfeeding support through several face-to-face contacts [26].

The integration of postnatal lactation support within the CPNP therefore warrants investigation as it may provide an opportunity to increase vulnerable mothers’ access to skilled lactation support and thereby improve duration and exclusivity of breastfeeding. One example of this approach is the Parkdale Parents’ Primary Prevention Project (5Ps), a CPNP site in Toronto. Through additional charitable funding, the 5Ps CPNP offers its clients free postnatal lactation support as a program enhancement. This includes in-home International Board Certified Lactation Consultant (IBCLC) services and double electric breast pumps. A qualitative study showed that clients highly valued the lactation support and reported that it helped to mitigate a variety of breastfeeding challenges [27].

To further build the evidence-base for the integration of postnatal lactation support within the CPNP, this research aimed to describe infant feeding practices among 5Ps CPNP clients, including breastfeeding (initiation, duration and exclusivity to 6 months), vitamin D supplementation of breastfed infants and timing of introduction to solids. Additional aims were to determine whether breastfeeding outcomes at 6 months differ based on maternal sociodemographics and food insecurity and assess utilization of the lactation support program.

## Methods

### Study population

This was a prospective study conducted at the 5Ps CPNP, a Toronto CPNP site implemented by Parkdale-Queen West Community Health Centre. The 5Ps CPNP has been operating for over 25 years and serves families in the South West area of Toronto. Services at the weekly prenatal program are free-of-charge and include group education workshops (e.g. prenatal nutrition, labour and delivery, newborn care); individualized support from public health nurses and dietitians (e.g. health/nutrition counselling); community referrals (e.g. public health programming, social work/counselling, settlement services); snacks; two public transit tokens; CDN$10 grocery store gift card; self-serve food bank; and childcare. Public health staff provide education on breastfeeding benefits and techniques approximately every 4–6 weeks as part of the annual 5Ps CPNP group education curriculum. The 5Ps CPNP is open to all women living within the catchment area, which include densely populated, ethnically diverse, low-income neighbourhoods. Women can enroll at any time during pregnancy and may attend the weekly program as often as they wish prenatally. Clients also have access to additional services provided by Parkdale-Queen West Community Health Centre and partner agencies, including a variety of individual and group services for new parents.

All women who registered in the 5Ps CPNP as of August 17, 2017 were eligible for the study with the exception of those who participated in our qualitative study [27]. Potential study participants were identified through the 5Ps CPNP registration list maintained by program staff. Women who had more than one pregnancy within the study timeframe were only included once. Study participants were recruited at any point prenatally and provided written informed consent for enrollment after the first author explained study details in-person during programming or by telephone. Professional interpreters were used for all non-English speaking participants. This study was approved by the Office of Research Ethics at the University of Toronto.

### Description of the postnatal lactation support program

Using charitable donations, the 5Ps CPNP provides postnatal lactation support to its clients in addition to the abovementioned comprehensive set of prenatal services. This lactation support program was designed and implemented by 5Ps CPNP staff and includes three components: i) a gift package of breastfeeding and infant care supplies (e.g. nursing pillow/diapers); ii) IBCLC visits within 48 h of referral; and iii) a double electric breast pump. The 5Ps CPNP program assistant provides verbal and written information regarding postnatal lactation services to eligible clients (those who attend the 5Ps CPNP a minimum of three times) when they receive the gift package prenatally. Around the client’s due date, the program assistant telephones them to inquire about the birth and to offer a referral for IBCLC services. Since clients are aware of the lactation support services prenatally, they may also telephone the program assistant after delivery to request IBCLC services. Uptake of IBCLC services is therefore driven by client needs and their interest in accessing the service. IBCLCs provide personalized breastfeeding support (e.g. assessment, education, management of breastfeeding concerns) through visits carried out in-hospital, in the client’s home or at the 5Ps CPNP office, depending on client preference. A second consultation is arranged at the client’s request and the discretion of the IBCLC. Clients with more challenging cases may receive a third IBCLC visit with approval from the 5Ps CPNP program coordinator. During the home visit, IBCLCs may provide clients with a double electric breast pump (Medela™ Pump in Style), demonstrate how to use it and advise on best practices for collecting and storing breastmilk. Clients may also receive a breast pump on the recommendation of a public health nurse or midwife. If needed, IBCLCs provide herbal galactagogues (herbal products used to increase breastmilk supply such as fenugreek) and lactation aids (e.g. supplemental nursing system; a method of providing extra breastmilk or formula by tube while infants feed at the breast). IBCLCs also provide guidance to clients to seek out a prescription for Domperidone (e.g. a medication used to increase breastmilk supply) from a physician, if necessary.

### Data collection

Maternal sociodemographics were self-reported by participants and collected by the first author at 2 weeks postpartum (Supplementary Table 1). Data included age, length of time in Canada, education, single parent status, number of children, household income and ethnicity. Participants self-identified their ethnicity from a standardized list of geographically-based categories developed and utilized by investigators of a large cohort study based out of Toronto [28]. As is done in the CCHS, household income was recorded as above or below the 2016 Statistics Canada size-adjusted Low-Income Cut-Off [29].

To assess food insecurity over the previous 12 months, the validated 18-item CCHS Household Food Security Survey Module (HFSSM) was administered at 6 months postpartum [30, 31]. According to Health Canada, the spectrum of household food insecurity includes worrying about running out of food and/or a food access problem (marginal), compromising food quality/quantity (moderate), and food deprivation (severe), each of which are related to financial constraints [30–32].

By telephone (or in-person during programming), at 2 weeks and 2, 4 and 6 months postpartum (+/− two weeks), the first author administered a standardized and validated infant feeding questionnaire used previously by our group (Supplementary Table 1) [33, 34]. During the first telephone call, participants were asked whether their infant received any formula in-hospital. At each data collection time point, participants were asked whether they were providing breastmilk (by breast or bottle), infant formula and any other liquids (e.g. cow’s milk, water, tea) and the average frequency of provision in the past two weeks. Those who discontinued breastfeeding were asked to recollect the last date they provided breastmilk. Details on supplemental vitamin D were collected as well as the date solids were introduced and the first solid food(s) provided.

Data on participant utilization of the lactation support program were extracted from electronic records maintained by the program and included whether participants had an IBCLC consult (yes/no), the timing to the first IBCLC visit postpartum (days), the number of visits with an IBCLC and whether they received a breast pump (yes/no).

### Statistical analyses

In order to thoroughly examine infant feeding practices at the 5Ps CPNP, we aimed a priori to recruit 200 participants since there are approximately 100 births to program clients annually. Data were analyzed using SAS Version 9.4 (SAS Institute).

Maternal sociodemographics were reported using descriptive statistics. These included age (years), number of years in Canada (born in Canada/< 1 year/1–3 years/≥3 years), education (<high school/high school/post-secondary), single parent (yes/no), number of children (first-time mother/≥1 child), household income (above Low-Income Cut-Off/below Low-Income Cut-Off/prefer not to answer or do not know) and ethnicity (East Asian/African/European/South Asian/Latin American/Caribbean/Southeast Asian/West Asian/North American Aboriginal/prefer not to answer or do not know). Household food insecurity was classified as yes/no and as secure/marginal/moderate/severe.

Participants were classified as yes/no for any breastfeeding and for EBF at each time point. The term “breastfeeding” was used to describe breastmilk (fresh or frozen) fed at the breast or by supplemental nursing system/bottle. EBF was defined as the provision of only breastmilk with no additional fluids or foods except for vitamins/minerals and medicine [10]. In compliance with WHO and Health Canada guidance that infants are ready for solids around 6 months of age, participants were considered EBF at the 6-month time point if solids were introduced within the previous two weeks and they were otherwise providing only breastmilk. As recommended by Greiner (2014), EBF was reported “at” each data collection time point (e.g. “at 6 months”) and “for” each time interval (e.g. “from birth to 6 months” and “from hospital discharge to 6 months”) [35].

Breastfed infants were classified as yes/no for vitamin D supplementation at each time point. Timing of the introduction to solids was based on the first reported date infants received solid foods. Early introduction was defined as starting solids more than two weeks before 6 months. Infants’ first solid food(s) were classified as iron-rich if they included fortified infant cereal, meat, eggs or beans/legumes [36].

Associations between maternal characteristics and breastfeeding outcomes (any breastfeeding at 6 months, EBF to 6 months post-discharge, EBF at 6 months) were assessed using Chi-square tests (categorical variables) and t-tests (continuous variables). Given the high heterogeneity of ethnicities in our cohort and thus small sample size in most ethnicities, we did not assess the relationship of ethnicity with breastfeeding outcomes.

Data on utilization of the lactation support program were reported using descriptive statistics.

## Results

### Study participants

**Fig. 1:**
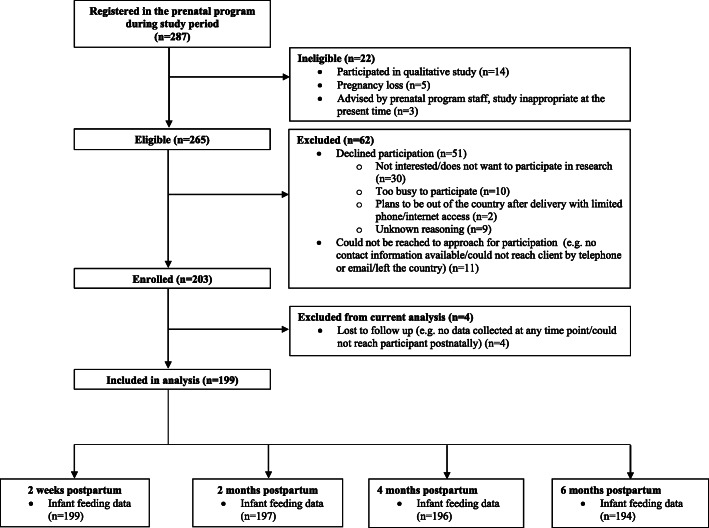
Participant flow diagram

During the study period, 287 women registered in the 5Ps CPNP (Fig. 1). Of these, 14 were ineligible based on prior participation in our qualitative study [27]. Five became ineligible due to pregnancy loss and 5Ps CPNP staff advised that study participation would be inappropriate for three women. Two hundred and three eligible mothers enrolled in the study. Four were lost to follow up as they could not be reached for any data collection following five contact attempts on various days of the week and at different times of the day at each data collection point. Recruitment was completed in January 2020.

Mean maternal age of study participants was 32 years, 91% were born outside of Canada and 55% reported a household income below the Low-Income Cut-Off (Table 1). Overall, 55% reported household food insecurity over the previous 12 months; 13, 32 and 10% reported marginal, moderate or severe food insecurity, respectively.

**Table 1:** Characteristics of study participants (*n* = 199)

Characteristics	n	%
**Age, average years (SD)**	32.3 (5.3) [*n* = 196]	–
**Number of years in Canada**		
Born in Canada	17	9
< 1 year	18	9
1- < 3 years	45	23
≥ 3 years	117	59
Missing	2	
**Completed education**		
< High school	16	8
High school	55	28
Post-secondary	126	64
Missing	2	
**Single parent**		
Yes	59	30
No	137	70
Missing	3	
**Number of children**		
First-time mother	97	49
≥ 1 child	99	51
Missing	3	
**Household income**^**a**^		
Below Low-Income Cut-Off	108	55
Above Low-Income Cut-Off	71	36
Prefer not to answer/Don’t know income^b^	18	9
Missing	2	
**Food insecurity**		
Yes	106	55
No	87	45
Missing	6	
**Level of food insecurity**		
Secure	87	45
Marginal	26	13
Moderate	61	32
Severe	19	10
Missing	6	
**Ethnicity**		
East Asian	82	42
African	23	12
European	12	6
South Asian	28	14
Latin American	17	9
Caribbean	15	8
Southeast Asian	7	4
West Asian	9	5
North American Aboriginal	2	1
Prefer not to answer/Don’t know^c^	2	1
Missing	2	

Percentages reflect proportion of non-missing data; percentages might not add to 100 due to rounding

*SD* standard deviation

^a^To determine whether or not a participant was living above or below the Low-Income Cut-Off, the 2016 Statistics Canada size-adjusted Low-Income Cut-Off was used

^b^This category is composed of 4 participants who preferred not to respond to the question and 14 who reported that they did not know their household income

^c^This category is composed of 2 participants who reported that they did not know their ethnicity or preferred not to report their ethnicity

Professional interpreter services in eight different languages were used to communicate with 14 study participants.

### Infant feeding practices

#### Breastfeeding practices

**Fig. 2:**
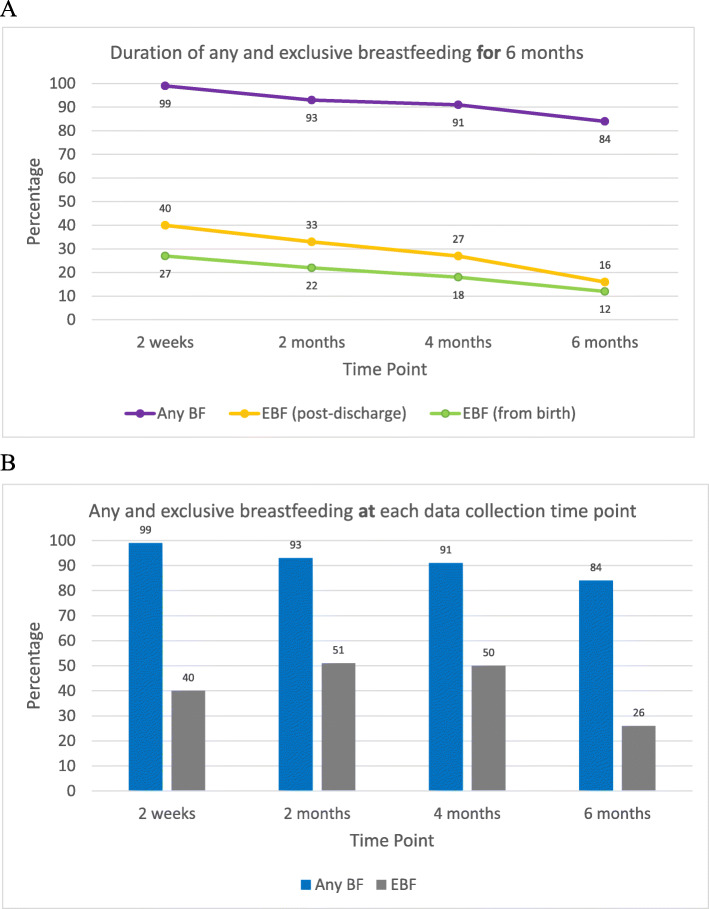
Breastfeeding practices among vulnerable women enrolled in a CPNP site offering enhanced postnatal lactation support. A, Proportion of infants breastfeeding and exclusively breastfeeding “for” 6 months. B, Proportion of infants breastfeeding and exclusively breastfeeding “at” each data collection time point. BF = breastfeeding; EBF = exclusive breastfeeding; CPNP=Canada Prenatal Nutrition Program

All study participants initiated breastfeeding. Sixty percent reported that their infant received formula supplementation in-hospital. Continued breastfeeding rates were high with 84% of participants still providing breastmilk at 6 months (Fig. 2). Of the 32 participants who stopped breastfeeding, 15/32 (47%) stopped before 2 months postpartum, 5/32 (16%) between 2 and 4 months, and 12/32 (38%) between 4 and 6 months.

Fifty percent of participants were EBF at 4 months, and 26% at six months (Fig. 2). We found lower proportions of continued EBF for 2, 4 and 6 months post-hospital discharge, and even lower proportions of EBF when taking into account in-hospital formula provision.

#### Vitamin D supplementation

Among participants providing breastmilk at 2 weeks, 2, 4 and 6 months, 76, 88, 84 and 80% were providing vitamin D supplementation, respectively.

#### Introduction to solids

Within the study period, 84% of infants were introduced to solids, 66% of whom received an iron-rich food source as their first food. The majority were introduced to solids before 6 months (52%), with most starting at 4 months (14%) or 5 months (34%). Only 31% started solids at 6 months, as is recommended in Canada.

### Maternal sociodemographics and food insecurity and breastfeeding

No statistically significant differences were found for any assessed breastfeeding outcomes based on parity, number of years in Canada, single-parenting, household income or age. However, a higher proportion of women who were not providing any breastmilk at 6 months reported food insecurity (*p* = 0.0140) (Table 2). In addition, high school education or less was associated with lower rates of EBF for six months post-discharge (*p* = 0.0344) (Table 2). Differences between maternal sociodemographics and EBF at six months showed similar results (Supplementary Table 2).

**Table 2:** Univariate analysis results: Relationship between maternal sociodemographics and food insecurity and 6-month breastfeeding outcomes

	Any Breastfeeding at 6 Months			Exclusive Breastfeeding for 6 Months Post-hospital Discharge		
	No No./Total (%)	Yes No./Total (%)	***p***-value	No No./Total (%)	Yes No./Total (%)	p-value
**Education**						0.0344*
≤ High school	11/28 (39)	60/165 (36)	0.7669	64/163 (39)	6/31 (19)	
Post-secondary	17/28 (61)	105/165 (64)		99/163 (61)	25/31 (81)	
**Food insecurity**						0.0510
Yes	22/29 (76)	84/164 (51)	0.0140*	93/161 (58)	12/31 (39)	
No	7/29 (24)	80/164 (49)		68/161 (42)	19/31 (61)	
**Number of children**						0.2013
First-time mother	9/28 (32)	85/164 (52)	0.0541	83/162 (51)	12/31 (39)	
≥ 1 child	19/28 (68)	79/164 (48)		79/162 (49)	19/31 (61)	
**Years in Canada**						0.4104
< 3 years	11/28 (39)	51/165 (31)	0.2831	54/163 (33)	7/31 (23)	
≥ 3 years	13/28 (46)	101/165 (61)		96/163 (59)	20/31 (65)	
Born in Canada	4/28 (14)	13/165 (8)		13/163 (8)	4/31 (13)	
**Single parent**						0.5477
Yes	9/28 (32)	48/164 (29)	0.7583	47/162 (29)	11/31 (35)	
No	19/28 (68)	116/164 (71)		115/162 (71)	20/31 (65)	
**Household income**						0.5898
Below Low-Income Cut-Off	17/24 (71)	90/152 (59)	0.2784	91/149 (61)	15/27 (56)	
Above Low-Income Cut-Off	7/24 (29)	62/152 (41)		58/149 (39)	12/27 (44)	
**Age** (mean, SD)^a^	32.1 (6.17)	32.4 (5.10)	0.8274	32.4 (5.29)	31.9 (5.19)	0.6791

Data analyzed using Chi-square tests for categorical variables and T-tests for continuous variables. Participants with missing breastfeeding outcome data were excluded from these analyses (n = 4 for any breastfeeding at 6 months and *n* = 3 for exclusive breastfeeding for 6 months post-hospital discharge). The total number may vary between cells depending on missing sociodemographic data. *p < 0.05

^a^Missing age data for three participants

### Postnatal lactation support program utilization

Lactation support program utilization was high, with 75% of study participants having received at least one IBCLC visit; 95% of these received a breast pump (Table 3). The median number of days postpartum to the first IBCLC visit was 5 (interquartile range: 3–10). For the number of IBCLC visits, 35, 34 and 6% of study participants had 1, 2 or 3 visits, respectively.

**Table 3:** Lactation support program utilization (*n* = 199)

Lactation support program	n	%
≥ 1 IBCLC visit	150	75
IBCLC and pump	143	72
Days postpartum to first IBCLC visit, median (IQR)	5 (3–10)	–
Number of visits with IBCLC		
0	49	25
1	70	35
2	68	34
3	12	6

*IBCLC* International Board Certified Lactation Consultant, *IQR* interquartile range

## Discussion

The CPNP aims to promote and support breastfeeding among vulnerable Canadian women, but there is currently no formally established framework or funding for sites to provide postnatal lactation support. Through additional charitable donations, the 5Ps CPNP site in Toronto offers its clients free, in-home postnatal lactation support. In this prospective study, we investigated infant feeding practices and utilization of the lactation support program at the 5Ps CPNP. Our findings indicate that breastfeeding initiation and continued breastfeeding for 6 months were high, although few participants EBF for 6 months. We also found high uptake of the lactation support program and few differences in assessed breastfeeding outcomes based on maternal characteristics.

All women in this study initiated breastfeeding and 84% were still breastfeeding to some degree at 6 months. This is in comparison to national CCHS data which show that 91% women initiate breastfeeding and almost half stop by six months [11, 36, 37]. The only national CPNP impact evaluation, conducted in 2002–2006 (*n* = 48,184), found that 89% of participants initiated breastfeeding, but 60% stopped by 6 weeks postpartum [19, 20]. Further data on breastfeeding duration and exclusivity among CPNP participants are not available. EBF for six months in Canada has been reported by the CCHS as 34% [11]. This value is higher than the EBF rates in our study (“at” 6 months [26%] and “for” 6 months from birth [12%] and post-discharge [16%]). Among food insecure women in the CCHS, EBF rates are lower than the national data and more comparable to our sample of vulnerable women [15]. It is important to keep in mind that measuring and reporting EBF is complex. Despite calls for clear and consistent EBF indicators over the past two decades, it remains challenging to interpret and compare EBF practices between studies as there is variation in EBF rates based on the definitions used (e.g. “at” 6 months or “for” 6 months) and how data are collected (e.g. retrospectively or prospectively) [35, 38–41]. For example, in comparison to the prospective data collection in our study, CCHS breastfeeding data are cross-sectional and collected retrospectively from respondents who gave birth in the five years prior to the survey [42]. Nonetheless, gaps remain between study participants’ EBF practices and public health recommendations, but we did find high breastfeeding initiation and continuation among a group of vulnerable women exhibiting risk factors for low breastfeeding rates, such as low income, single-parenting and food insecurity [14, 15, 42]. It is likely that a combination of maternal commitment to breastfeeding and access to skilled lactation support contributed to the very high rates of continued breastfeeding, but results from our observational study cannot determine causality and we did not collect data on feeding intentions or breastfeeding self-efficacy. To further inform efforts to provide postnatal lactation support to vulnerable women through the CPNP, priority areas of research include a trial to examine the effectiveness of providing in-home lactation support on breastfeeding outcomes. The use of mixed-methods implementation research should be considered to help evaluate and guide the feasibility and scale up of this intervention [43, 44].

We found high uptake of the lactation support program, with three-quarters of study participants receiving at least one IBCLC consult, and approximately half of these accessing the second IBCLC visit. The median time to the first visit was 5 days postpartum, which may reflect the proactive offering of services through prenatal communication and early postpartum follow up by program staff. Skilled lactation support in the first two weeks postpartum is known to be critical for the management of breastfeeding difficulties [21, 45]. Our qualitative work investigating mothers’ experiences with the 5Ps CPNP lactation support program found that the lactation services were highly valued and helped address physical, practical and self-efficacy challenges related to breastfeeding [27]. These findings are encouraging given the 5Ps CPNP’s mandate for reducing inequities in service provision for vulnerable women in the perinatal period. Canadian guidelines acknowledge the importance of skilled lactation support in the community for new mothers and suggest direct assessment and follow up by skilled lactation professionals [9, 37], but private IBCLCs are not covered by provincial health insurance in Ontario and thus are typically only accessible to higher-income women. Free community breastfeeding services are typically offered reactively and are harder to access by vulnerable women [27]. Together, the data from our current study and our qualitative work suggest that a demand exists for skilled breastfeeding support among vulnerable women and that such services can be delivered through a CPNP site. Further research using implementation science approaches is needed to understand the feasibility and appropriateness of the integration of postnatal lactation support within CPNP sites [46]. The current context of the CPNP creates a foundation for supporting breastfeeding initiation among vulnerable women by providing an established social support structure, links to multiple community services to reduce life stresses and strong prenatal breastfeeding promotion. This existing comprehensive community program could therefore be enhanced to provide continued postnatal lactation support in a continuum-of-care model, as was done at the 5Ps CPNP. These results respond to recent calls to strengthen the evidence for leveraging existing community programs to increase access to lactation support for vulnerable women [47].

Women in this study experienced multiple vulnerabilities known to be associated with reduced breastfeeding, yet we found few differences in assessed breastfeeding outcomes at 6 months based on maternal sociodemographics. However, given the distribution of our breastfeeding data we were unable to conduct multivariable analyses. Our univariate analyses suggest women with high school education or less and living with household food insecurity may face greater barriers to breastfeeding, despite having access to postnatal lactation support. This is consistent with research demonstrating that education is a social determinant of health often associated with breastfeeding and with evidence that household food insecurity negatively influences breastfeeding practices, including a recent study using CCHS data which showed that household food insecurity was associated with a shorter duration of EBF [14, 15, 42]. Although breastfeeding contributes to infant food security and is protective of household resources, household food insecurity may be a barrier to continued and/or exclusive breastfeeding due to stress associated with food insecurity and mothers’ concerns regarding the quantity and quality of their breastmilk [16, 48, 49]. Food insecurity may therefore be an under-recognized barrier to breastfeeding among vulnerable populations in Canada and around the world [50, 51]. In Canada, approximately 16% of households with children less than 18 years of age experience food insecurity [32]. Among study participants, 55% reported household food insecurity. As we lacked statistical power to examine severity of household food insecurity in relation to breastfeeding outcomes and to conduct multivariable analyses, further research on food insecurity and breastfeeding outcomes among vulnerable women in Canada is warranted.

The 5Ps CPNP lactation support program, and the national CPNP in general, focuses on providing community-based support, but the use of formula in-hospital is a predictor of breastfeeding outcomes, especially among vulnerable women [52–54]. In-hospital formula use among study participants was common (60%) and higher than provincial data from Ontario (43%) [55]. Systematic review and meta-analysis evidence suggests a need to strengthen the delivery of breastfeeding support interventions in a combination of settings (e.g. in-hospital and in the community) to improve breastfeeding outcomes [56]. Thus, at the community-level, programs such as the 5Ps CPNP lactation support program may consider targeting the early-postpartum time period more explicitly and advocating for the increase of Baby-Friendly Initiative (BFI) designations [57, 58].

We investigated adherence to additional Health Canada infant feeding guidelines that pertain to breastfeeding (i.e. vitamin D supplementation for breastfed infants and introduction of solids) since Canadian data among vulnerable women are limited. We found over three-quarters of breastfed infants received vitamin D supplementation throughout the first 6 months, which is similar to rates among breastfed infants in the Alberta Pregnancy Outcomes and Nutrition (APrON) cohort and among EBF infants in the CCHS [42, 59]. Vitamin D supplementation among breastfed infants does not appear to be a concern among the vulnerable group of women in our study. Solid foods were introduced before 6 months by 52% of study participants, as is common in Canada [59, 60]. Early introduction of solids has been shown to undermine breastfeeding [61]. We did not collect data on the reason for the timing of solids introduction. Further research on this issue is needed, including how health care providers counsel parents on introducing solids, how parents interpret this guidance and what factors play a role in their decision to introduce solids. Among infants who were introduced to solids, 66% received an iron-rich first food, as recommended. Although we did not collect additional information on how long the first food was provided or when another, potentially iron-rich food was given, it is important to know that iron deficiency and iron deficiency anemia are still concerns in Canada, particularly among immigrants and refugees [62, 63].

To our knowledge, this is the first study to provide detailed data on infant feeding practices, including breastfeeding duration and exclusivity, among vulnerable women enrolled in a CPNP site. Strengths of this study include the prospective collection of infant feeding data, which helps to reduce recall bias [64], and the collection of implementation data (e.g. utilization of the lactation support program). Another strength is the high enrollment and retention rates, which are likely a result of the first author attending the weekly prenatal program throughout recruitment and data collection in order to become embedded within the program and to develop trust and rapport with participants [65]. Finally, the use of professional interpreters enabled participation of non-English speaking women.

In terms of limitations, we do not have data on the characteristics of eligible 5Ps CPNP clients who did not participate in our study, but our study sample characteristics are comparable to the sociodemographic profile of 5Ps CPNP clients based on program registration records. The 5Ps CPNP serves an ethnically diverse population, which was reflected in our study sample. However, this precluded us from analyzing the association between ethnicity and breastfeeding outcomes due to the predominance of one ethnic group and small sample sizes among remaining categories. Social desirability bias is another potential limitation, although high reporting of formula use suggests this was minimized and participants did not feel pressured to over-report breastfeeding practices. Our food insecurity data was limited by the fact that the HFSSM measures food insecurity over the previous 12 months, so it is possible that participants’ food insecurity experience might not have been concurrent with our 6-month postnatal data collection time period. We were also unable to conduct multivariable regression modelling to investigate associations between maternal characteristics and breastfeeding outcomes due to limited variability in breastfeeding outcomes in our sample. Due to the high uptake of the lactation support program and high breastfeeding rates, we also had limited power to examine associations between lactation support program utilization and breastfeeding outcomes. Additionally, findings are specific to the 5Ps CPNP, but they suggest that skilled lactation support can be delivered through a CPNP site, with high uptake by clients. This study is one step within a larger research program evaluating the potential for the CPNP to serve as a mechanism to increase access to lactation support for vulnerable women, and in doing so, reduce breastfeeding disparities.

## Conclusion

Among vulnerable women enrolled in the 5Ps CPNP with access to enhanced postnatal lactation support, we found high uptake of lactation services and strong adherence to many recommended infant feeding practices, including breastfeeding initiation, continued breastfeeding for 6 months and vitamin D supplementation for breastfed infants. Areas for improvement include the timing of introduction of solids and EBF for 6 months. This study provides initial evidence that postnatal lactation support can be delivered through a CPNP site, with high uptake by clients and corresponding high rates of breastfeeding. Further research using mixed-methods implementation science approaches is needed to understand the feasibility and effectiveness of the integration of postnatal lactation support within CPNP sites.

## Supplementary Information

**Additional file 1.** Data collection timeline.

**Additional file 2.** Univariate analysis results: Relationship between maternal sociodemographics and food insecurity and exclusive breastfeeding at 6 months.

## Data Availability

The datasets generated and/or analysed during the current study are not publicly available in order to protect participant anonymity and confidentiality.

## Abbreviations

5Ps: Parkdale Parents’ Primary Prevention Project
CCHS: Canadian Community Health Survey
CPNP: Canada Prenatal Nutrition Program
EBF: Exclusive breastfeeding
HFSSM: Household Food Security Survey Module
IBCLC: International Board Certified Lactation Consultant
WHO: World Health Organization
