# Supplementary material for: Breastfeeding rates are high in a prenatal community support program targeting vulnerable women and offering enhanced postnatal lactation support: a prospective cohort study
Source: Int J Equity Health. 2021 Mar 3;20:71. doi: 10.1186/s12939-021-01386-6 (PMC7931510; doi:10.1186/s12939-021-01386-6)
Supplement: Supplementary file 1 — Additional file 1. Data collection timeline. [file 12939_2021_1386_MOESM1_ESM.pdf]

Supplementary Table 1. Data collection timeline

Study Instrument	Postnatal Data Collection			
	2 weeks	2 months	4 months	6 months
Maternal Sociodemographics Questionnaire[a]	X			
Infant Feeding Questionnaire	X	X	X	X
Food Security Questionnaire[b]				X

[a]Maternal sociodemographics include age, length of time in Canada, education, single parent status, number of children, ethnicity and household income

[b]The 18-item Canadian Community Health Survey Household Food Security Survey Module was used to measure household food insecurity
