# Supplementary material for: Breastfeeding rates are high in a prenatal community support program targeting vulnerable women and offering enhanced postnatal lactation support: a prospective cohort study
Source: Int J Equity Health. 2021 Mar 3;20:71. doi: 10.1186/s12939-021-01386-6 (PMC7931510; doi:10.1186/s12939-021-01386-6)
Supplement: Supplementary file 2 — Additional file 2. Univariate analysis results: Relationship between maternal sociodemographics and food insecurity and exclusive breastfeeding at 6 months. [file 12939_2021_1386_MOESM2_ESM.pdf]

Supplementary Table 2. Univariate analysis results: Relationship between maternal sociodemographic characteristics and exclusive breastfeeding at 6 months

**Maternal Characteristics**	**Exclusive Breastfeeding at 6 months**		
	**No No./Total (%)**	**Yes No./Total (%)**	**p-value**
Education			
≤ High school	58/140 (41)	10/50 (20)	0.0067*
Post-secondary	82/140 (59)	40/50 (80)	
Food insecurity			
Yes	80/142 (56)	25/50 (50)	0.4388
No	62/142 (44)	25/50 (50)	
Number of children			
First-time mother	70/139 (50)	23/50 (46)	0.5969
≥ 1 child	69/139 (50)	27/50 (54)	
Years in Canada			0.3452
<3 years	45/140 (32)	15/50 (30)	
≥3 years	85/140 (61)	28/50 (56)	
Born in Canada	10/140 (7)	7/50 (14)	
Single parent			
Yes	40/139 (29)	17/50 (34)	0.4901
No	99/139 (71)	33/50 (66)	
Household income			
Below Low-Income Cut-Off	78/129 (61)	27/44 (61)	0.9161
Above Low-Income Cut-Off	51/129 (40)	17/44 (39)	
Age (mean, SD)[a]	32.6 (5.41)	31.6 (4.93)	0.2416

Data analyzed using Chi-square tests for categorical variables and T-tests for continuous variables. Participants with missing exclusive breastfeeding at 6 months data were excluded from these analyses (n=7). The total number may vary between cells depending on missing sociodemographic data. *p<0.05
[a]Missing age data for three participants.
